# Peripheral blood transcriptomics identifies cohesin–chromatin and immune dysregulation in tics and Tourette syndrome

**DOI:** 10.3389/fneur.2026.1730761

**Published:** 2026-04-16

**Authors:** Shrujna Patel, Velda X. Han, Brooke A. Keating, Hiroya Nishida, Jessica P. Hayes, Erica Tsang, Nader Aryamanesh, Lee L. Marshall, Shekeeb S. Mohammad, Russell C. Dale

**Affiliations:** 1 Kids Neuroscience Center, The Children's Hospital at Westmead, Faculty of Medicine and Health, University of Sydney, Sydney, NSW, Australia; 2 The Children's Hospital at Westmead Clinical School, Faculty of Medicine and Health, University of Sydney, Sydney, NSW, Australia; 3 Khoo Teck Puat-National University Children's Medical Institute, National University Health System, Singapore, Singapore; 4 Department of Brain and Neurosciences, Tokyo Metropolitan Institute of Medical Science, Tokyo, Japan; 5 Bioinformatics Group, Children's Medical Research Institute, University of Sydney, Westmead, Sydney, NSW, Australia

**Keywords:** chromatin, epigenetic, neurodevelopmental disorders, OCD (obsessive-compulsive disorder), RNA sequencing

## Abstract

**Introduction:**

Tic disorders and Tourette syndrome are neurodevelopmental disorders arising from interplay between genetic and environmental factors. Gene regulation, via chromatin and other epigenetic mechanisms, may provide a key link between gene-environment interactions, yet remains under-investigated in tic disorders.

**Methods:**

We performed bulk RNA sequencing of peripheral blood from 28 children with tic disorders (19 with Tourette syndrome, aged 6–16, median age 10 years, 18 (64%) males) compared to 20 matched healthy controls (aged 1–25, median age 11 years, 13 (65%) males). Differentially expressed genes (DEGs) were identified following false discovery rate (FDR) correction, and pathway enrichment was analyzed using Gene Set Enrichment Analysis (GSEA) based on Gene Ontology (GO) and Reactome databases. To assess convergence between peripheral and brain molecular changes, DEGs were compared with published post-mortem brain transcriptomic data from individuals with Tourette syndrome.

**Results:**

A total of 4,169 DEGs (FDR < 0.05) were identified in blood transcriptomic analysis, with 2,192 upregulated and 1,977 genes. The blood transcriptomic findings included upregulation of chromatin- and cohesin-related pathways, immune activation, and cell signaling, and downregulation of translational machinery, mitochondrial function, and DNA methylation. Comparison with post-mortem brain transcriptomic data revealed 30 overlapping genes, including 20 that were concordantly upregulated in both blood and brain, predominantly associated with immune, signaling, and cellular stress responses.

**Discussion:**

Our data points to gene regulation and chromatin biology as a nexus where genetic risk and environmental exposures converge in tic disorders and related neurodevelopmental disorders, and highlight epigenetic and immune-targeting therapies as promising avenues for disease modification.

## Introduction

Tic disorders, including Tourette syndrome, are the most common movement disorders affecting children (1). The DSM-V defines tics as “sudden, rapid, recurrent, nonrhythmic motor movement (motor tics) or vocalization (vocal or phonic tics)” (2). The clinical course is heterogeneous, with fluctuations in tic severity referred to as ‘waxing and waning'. Tics share a strong and complex neurobiological relationship with other neurodevelopmental conditions (3, 4), particularly obsessive–compulsive disorder [OCD; affecting 30%−50% of individuals with Tourette syndrome (5)] and attention-deficit hyperactivity disorder [ADHD; 50%−60% (5, 6)]. Anxiety and depression are also common, affecting about 30% of individuals (5), while autism spectrum disorder (ASD) is less commonly associated [4%−20% (7)]. Current treatments for tic disorders, such as antipsychotics, focus on managing symptoms and there are no disease-modifying treatments available.

Impaired cortico-striatal circuits with aberrant associated neurotransmitter function, particularly dopamine, have been implicated in the pathophysiology of tic disorders, though specific mechanisms remain unclear (8, 9). Twin and family studies confirm that tic disorders are heritable (10, 11), although only a small number of highly penetrant genetic variants have been identified (12–14). Genetic studies have implicated many synaptic, neuronal, and immune-related susceptibility genes (11, 15–17). As is the case for most common neurodevelopmental disorders, it is now widely accepted that tic disorders have a multifactorial etiology, involving interactions between many susceptibility genes plus environmental factors (1, 8). Adverse perinatal factors (e.g., preterm birth, impaired fetal growth, low birth weight) and maternal inflammation during pregnancy, including autoimmune disease, have been associated with increased risk of Tourette syndrome in offspring (18–22).

Indeed, there is a long-standing literature describing the role of immune and inflammatory mechanisms in the pathophysiology of tic disorders (4, 23, 24). Infections and stress are frequently reported as triggers for tic onset or symptom exacerbation (22, 25–28). In some cases, this presents as pediatric autoimmune neuropsychiatric disorders associated with streptococcal infections (PANDAS) (29, 30) or pediatric acute-onset neuropsychiatric syndrome (PANS) (31, 32), clinical phenotypes thought to reflect immune brain dysregulation. Immune dysfunction, such as proinflammatory cytokine profiles, changes in immune cell populations, and abnormal immunoglobulin concentrations have been found in individuals with Tourette syndrome (33–35).

Increasing attention has turned to gene regulation as a mechanism by which environmental factors, such as inflammation and stress, influence neurodevelopment (36–39). Epigenetic processes, including DNA methylation and histone modifications, regulate chromatin accessibility and gene expression, thereby shaping the transcriptomic landscape of brain and immune cells (40, 41). A post-mortem transcriptomic study of the striatum in Tourette syndrome found upregulation of immune-related genes and downregulation of synaptic and neuronal genes (42), and similar findings are described in OCD (43) and ASD (44, 45). However, transcriptomic studies in tic disorders remain scarce, and even fewer have examined peripheral tissues in pediatric cohorts. Peripheral blood provides an accessible window into systemic immune and regulatory processes implicated in tic disorders and may yield biomarkers that complement brain-based findings. To address this gap, we used bulk RNA sequencing of peripheral blood to explore gene expression changes and identify cellular pathways involved in tic disorders. We also compared our blood data with previously published Tourette syndrome brain RNA sequencing data (42, 45) to explore concordance between peripheral and brain transcriptomic profiles. Our study elucidates molecular mechanisms underpinning tic disorders and highlights novel therapeutic targets (such as epigenetic drugs) that may enable disease modification, addressing an unmet need in current treatment approaches.

## Materials and methods

### Participants

The tics/Tourette syndrome patient group included 28 children (< 18 years) from 27 families (including two siblings) referred with tics to a specialist clinic at the Children's Hospital at Westmead, Sydney. This clinic receives a referral enrichment of patients with tics, Tourette syndrome, and OCD, often with accompanying conditions such as ADHD. A standardized REDCap questionnaire was used to document all family medical history and medications (46). Diagnosis of tic disorders, Tourette syndrome, and other comorbidities was made according to DSM-V criteria. Children with comorbid ASD, or those with a PANS or PANDAS phenotype, were excluded. None of the patients had an infection in the 2 weeks before blood collection.

### Control selection

We recruited 20 healthy volunteers who had no neurodevelopmental or neuropsychiatric disorders, autoimmune diseases, severe allergic conditions, or infection in the 2 weeks before blood collection. Controls were sex-matched to patients and there was no statistically significant age difference between the groups.

### Sample collection

After gaining written informed consent, venous blood was collected directly into PAXgene™ (PreAnalytiX GmbH, Hombrechtikon, Switzerland) Blood RNA tubes (BD Biosciences) and stored at −80 °C until extraction and sequencing by the Australian Genome Research Facility (AGRF).

#### Bulk blood RNA sequencing (library preparation and sequencing)

Bulk RNA sequencing for the full cohort was performed on whole blood by AGRF. This workflow included RNA extraction, depletion of ribosomal RNA via hybrid capture (Illumina Ribo-Zero), and Illumina TruSeq (Illumina, Inc., San Diego, CA, USA) Stranded Total RNA Library Preparation (input 200 ng−1,000 ng of Total RNA). The stranded RNA samples were sequenced on the Illumina NovaSeq X (Illumina, Inc., Hayward, CA, USA) sequencing platform (2 x 150 base pairs) for a depth of 50 million paired end reads. Raw sequencing reads were first evaluated for quality using FastQC (v0.12.1) and subsequently trimmed with Trimmomatic - Java-based command-line tool (75) (v0.39) to remove adapter sequences and low-quality bases. The cleaned sequence reads were aligned against the *Homo sapiens* genome (Build version hg38), and the STAR aligner (v2.7.10b) was used to map unique reads to the genomic sequences (47). Duplicate reads were identified and marked using Picard MarkDuplicates (v3.1.1), and gene-level read counts were quantified using featureCounts (v2.0.1).

### Bioinformatic analysis

RNA sequencing data were analyzed in the R statistical environment (48) with *tidyverse* (49). Filtering and normalization steps were first performed, followed by removal of unwanted variation, via the *remove unwanted variation* (RUV) package (50). In this study, *k* = 10 (factor of unwanted variation) was used to remove genes with minimal differential expression relative to negative control genes. For linear modeling, the *limma* package was used and the *p*-values were calculated using the empirical Bayes (*eBayes*) method (51). False discovery rate (FDR) correction was applied to obtain adjusted *p*-values, and significant differentially expressed genes (DEGs) were defined as those with FDR < 0.05.

#### Gene set enrichment analysis (GSEA)

Pathway-level enrichment was prioritized to identify cumulative effects of gene expression changes rather than focusing on individual DEGs. The genes were ranked based on their sign (logFC) x –log10 *p*-value scores (52, 53). Enriched gene sets were identified using a running sum statistic (adjusted *p*-values) and statistical significance assessed by FDR. Pathway enrichment analysis was performed via Gene Set Enrichment Analysis (GSEA) to obtain enriched Gene Ontology (GO) and Reactome pathways using the *fgsea* package. Significant GO pathways (FDR < 0.05) were further simplified using the *simplify* function in *clusterProfiler* (54). Significant pathways from GO and Reactome were ranked by normalized enrichment score (NES) and bar plots of the results were generated using *ggplot2*.

#### Tourette syndrome brain transcriptome analysis

To explore concordance between peripheral and brain transcriptomic profiles, we compared our blood DEGs with those identified in previously published Tourette syndrome brain RNA sequencing data (45) obtained with the authors' permission (42). The brain dataset included samples from the striatum of 9 Tourette syndrome cases (median age 52, range 29–84 years, 5 (56%) males) and 9 healthy controls (median age 52, range 4–60 years, 5 (56%) males). The full demographic data is in the Supplementary material of the original study [See their Supplementary Table 2 (42)]. Differentially expressed genes (FDR < 0.05) were compared between the two datasets to identify overlapping genes, including concordant and discordant directions of expression.

### Ethics approval

Ethical approval was granted by the Sydney Children's Hospitals Network Human Research Ethics Committee (HREC/18/SCHN/227, 2021/ETH00356). All families provided written informed consent for the study.

## Results

### Cohort description

The tic/Tourette syndrome patient group included 28 children from 27 families (including two siblings), aged 6–16 years (median age 10 years at time of sampling, 18 (64%) males). All 28 children had tics; 19 had Tourette syndrome and 9 had other tic disorders. Other comorbidities were present, including OCD (*n* = 16), ADHD (*n* = 9), anxiety (*n* = 15), sensory issues (*n* = 6), depression (*n* = 3), post-traumatic stress disorder (PTSD; *n* = 2), and intellectual disability (*n* = 1). None of the children had autism or autistic regression, and none fulfilled PANS/PANDAS criteria. The age of symptom onset ranged from 2–12 years (median 5 years). A family history of neurodevelopmental or neuropsychiatric disorders was reported in 15 mothers (tics = 2, OCD = 3, ADHD = 4, anxiety = 11, depression = 6, PTSD = 2, and postnatal depression = 2) and 12 fathers (tics = 4, OCD = 1, ADHD = 1, anxiety = 4, depression = 2, PTSD = 10). A history of autoimmune disease was present in four mothers and one father. Probands had a mean of 1.2 siblings, and 11 had one or more siblings with a neurodevelopmental or neuropsychiatric disorder. At time of sampling, 21 children were taking at least one medication, including fluoxetine (*n* = 8), clonidine (*n* = 6), fluvoxamine (*n* = 3), methylphenidate (*n* = 3), risperidone (*n* = 2), quetiapine (*n* = 1), escitalopram (*n* = 1), and mirtazapine (*n* = 1). The patients had a disease duration of 4 years (median 3, range 0–10 years) from symptom onset, during which time patients trialed a mean of 1.9 medications (median 2, range 0–5). The control group included 20 healthy volunteers, aged 1–25 years [median age 11 years at time of sampling, 13 (65%) males].

#### Peripheral blood RNA sequencing: principal component analysis and differentially expressed genes

**Figure 1:**
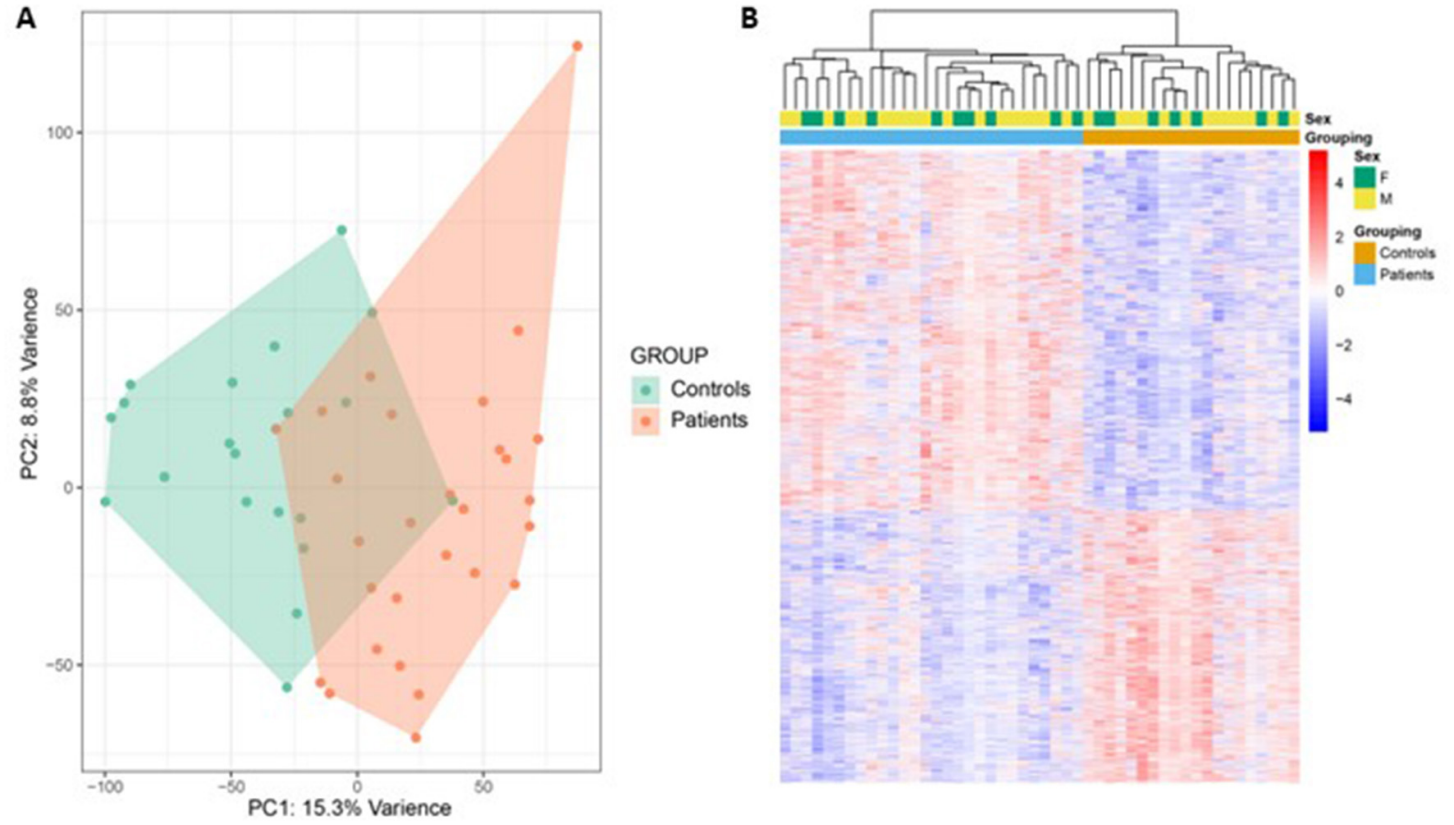
Peripheral blood bulk RNA sequencing principal component analysis and heatmap of differentially expressed genes. **(A)** Post RUVIII normalization principal component analysis (PCA) of bulk RNA sequencing shows some discrimination between patients and controls, with a degree of overlap. The x-axis represents principal component 1 (PC1), while the y-axis represents principal component 2 (PC2). **(B)** A heatmap of differentially expressed genes (DEGs) false discovery rate (FDR) < 0.05 shows clear visual discrimination of gene expression profiles between patients and controls. Upregulated genes are shown in red, downregulated genes are shown in blue. The figure legend displays *Z*-scores.

Bulk RNA sequencing was performed for the full cohort of patients and controls in the same batch. Post RUVIII normalization principal component analysis (PCA) showed partial discrimination between patients and controls (Figure 1A). There were 4,169 differentially expressed genes (DEGs) FDR < 0.05, with 2,192 genes upregulated, and 1,977 genes downregulated. A heatmap of DEGs showed clear visual discrimination between the patient and control groups (Figure 1B).

#### Peripheral blood RNA sequencing pathway analysis: upregulated GO and Reactome pathways

**Figure 2:**
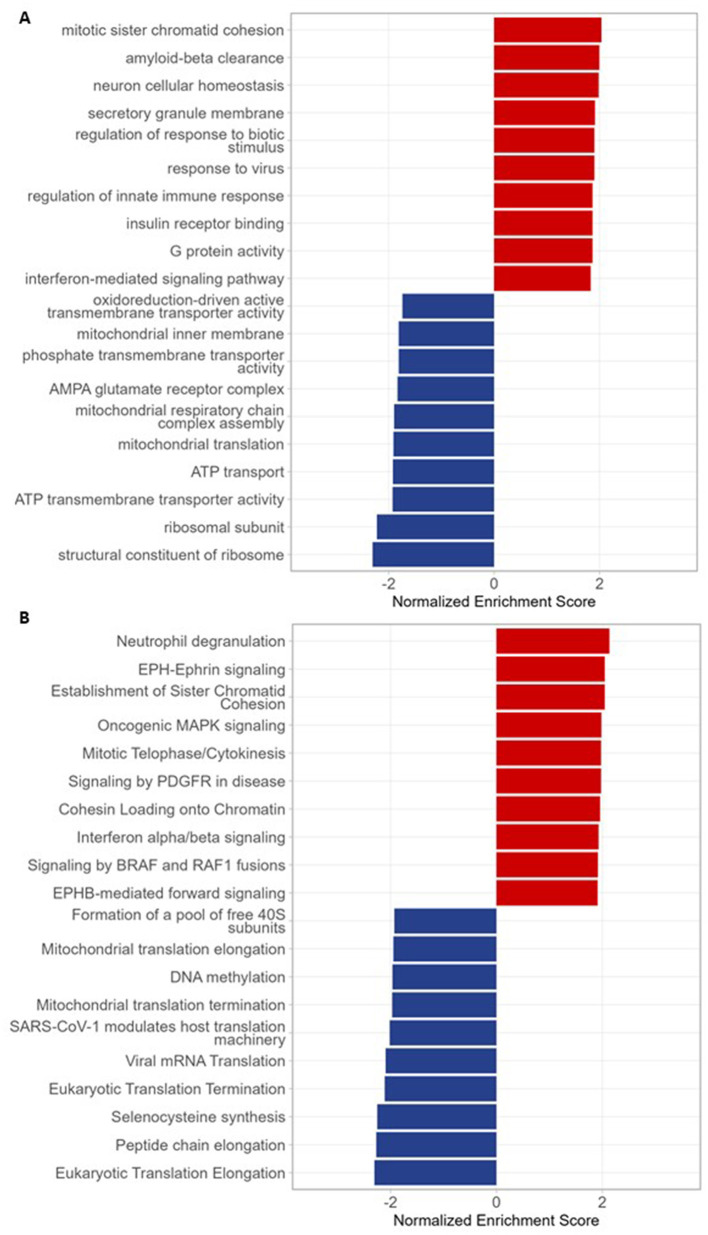
Peripheral blood bulk RNA sequencing Gene Set Enrichment Analysis (GSEA). **(A)** Bar plot of the top 10 Gene Ontology pathways in patients vs. controls (false discovery rate (FDR) < 0.05). The top 10 upregulated pathways (red) are related to chromatid cohesion, immune activation, and cellular signaling. The top 10 downregulated pathways (blue) are related to translational machinery (ribosome) and mitochondrial function. **(B)** Bar plot of top 10 Reactome pathways in patients vs. controls (FDR < 0.05). The top 10 upregulated pathways (red) are related to immune activation, cellular signaling, chromatid cohesion, and chromatin loading. The top 10 downregulated pathways (blue) are related to translational machinery (ribosome), protein synthesis, mitochondrial function, and DNA methylation.

The top upregulated GO pathway was ‘mitotic sister chromatin cohesion' (Figure 2A), enriched by genes that form the cohesin core complex (*RAD21, SMC3, STAG1, STAG2*), cohesin regulators (*NIPBL, PDS5B*), chromatin remodelers (*ATRX*) and histone variants (*MACROH2A1*), as well as genome stability (*SMC5)* and cell cycle genes (*RB1*; Table 1). Similar genes also enriched the ‘establishment of sister chromatid cohesion' and ‘cohesin loading on chromatin' pathways which were upregulated in Reactome (Figure 2B), indicating overall upregulation of cohesin and chromatin-related genes in this cohort.

**Table 1:** The top upregulated Gene Set Enrichment Analysis (GSEA) Gene Ontology pathway was ‘mitotic sister chromatid cohesion' (FDR < 0.05). It was enriched by 11 genes, which can be categorized into functional subclusters as follows.

Functional category	Genes	Main role
Cohesin core complex	*RAD21, SMC3, STAG1, STAG2*	Form the cohesin ring (RAD21 “clasp” with SMC proteins; STAG subunits determine variant complexes). Essential for chromatid cohesion, segregation, and transcriptional regulation
Cohesin regulators (loading, stabilization, establishment)	*NIPBL, PDS5B, ESCO1*	NIPBL = loads cohesin onto DNA; PDS5B = stabilizes cohesin on chromatin and regulates release; ESCO1 = acetyltransferase that establishes cohesion during S-phase
Chromatin remodelers and histone variants	*ATRX, MACROH2A1*	ATRX = chromatin remodeler involved in heterochromatin, telomere regulation, gene silencing; MACROH2A1 = histone variant regulating transcriptional repression and X-inactivation
Genome stability and DNA repair	*SMC5*	Member of the SMC5/6 complex; maintains genome integrity, homologous recombination, DNA repair
Cell cycle and tumor suppression	*RB1*	Encodes retinoblastoma protein (pRB), master regulator of the G1/S cell cycle checkpoint, integrates signaling and chromatin state to control proliferation

**Figure 3:**
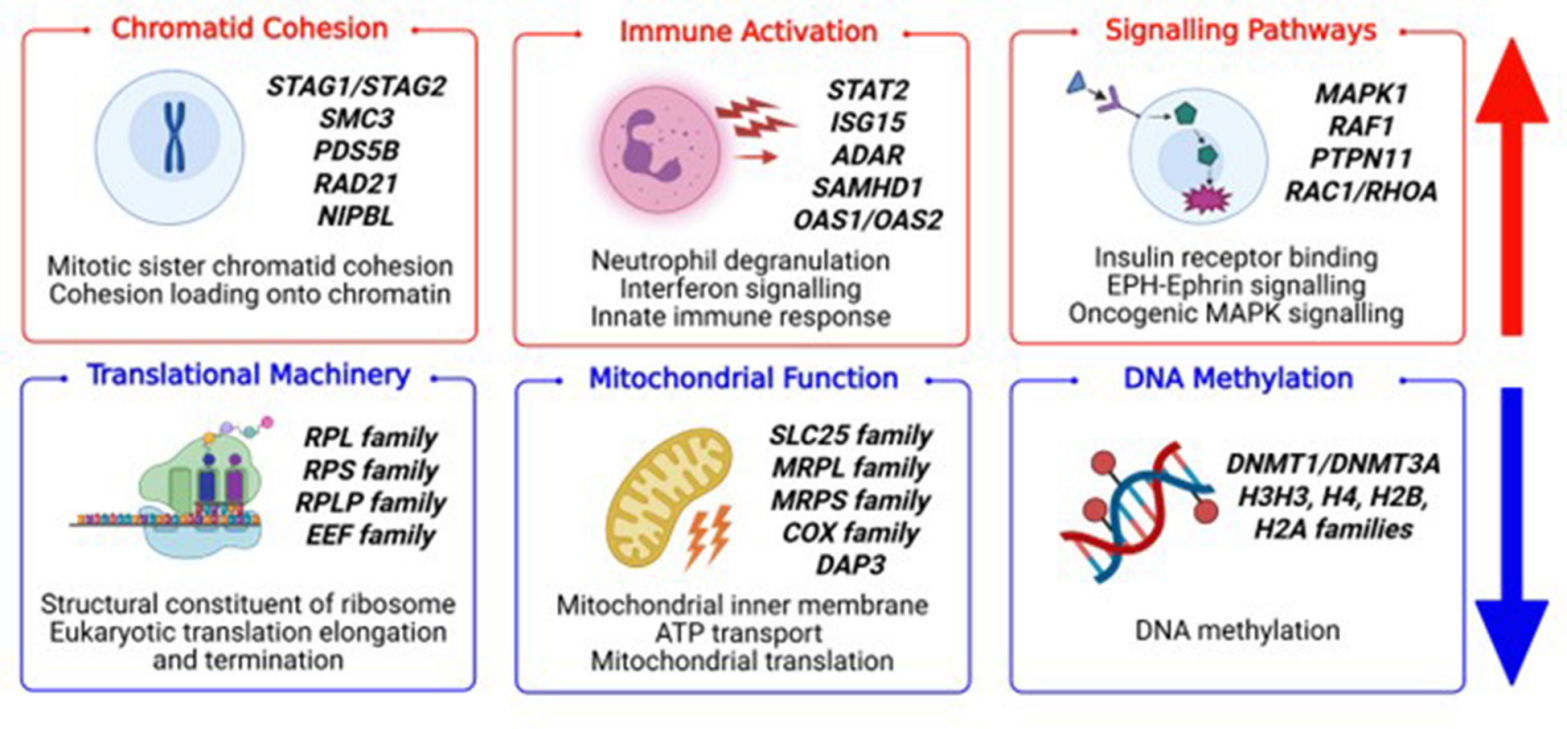
Summary of the Gene Set Enrichment Analysis (GSEA) of Gene Ontology and Reactome. The main upregulated pathways involved immune activation, chromatid cohesion, and cell signaling pathways. The main downregulated pathways were related to mitochondrial function, translational machinery, and DNA methylation. Illustrative genes enriching these pathways are highlighted in each panel. Figure created in BioRender.

The top upregulated Reactome pathway was ‘neutrophil degranulation' (Figure 2B), enriched by diverse immune receptors and signaling molecules, proteases, lysosomal proteins, cytoskeleton regulators, GTPases, metabolic enzymes, complement and innate effectors, as well as apoptosis, stress response, and other regulator genes (Table 2). In both GO and Reactome analyses, additional immune and cell-signaling pathways were upregulated, suggesting broad immune activation across multiple signaling cascades (Figures 2A, B, 3).

**Table 2:** The top upregulated Gene Set Enrichment Analysis (GSEA) Reactome pathway was ‘neutrophil degranulation' (FDR < 0.05). It was enriched by 151 genes, which can be categorized into functional subclusters as follows.

Functional category	Genes	Main role
Immune receptors and signaling molecules	*TNFRSF1B, FCGR2A, FPR1, FPR2, CXCR1, CXCR2, CD14, CD36, CD44, CD53, CD58, CD59, CD68, CD93, CEACAM1, CEACAM3, PECAM1, SIGLEC9, SIRPA, SIRPB1, NFAM1, NFKB1, ADGRE3, ADGRG3, FCER1G, ITGAX, MNDA, TICAM2, C5AR1*	Cell surface receptors, immune activation, phagocytosis, cytokine/chemokine signaling
Signaling kinases	*MAPK1, MAPK14, PRKCD*	Stress-activated MAP kinases and PKC family kinases driving inflammatory signaling, cytokine responses, and neutrophil activation
Proteases, peptidases and processing enzymes	*CTSS, CTSD, CTSA, CST3, MME, ADAM10, PRCP, QPCT, LTA4H, ANPEP, PLD1, DOK3, SERPINB1*	Proteolytic enzymes and inhibitors that balance lysosomal degradation, extracellular matrix remodeling, and inflammatory mediator control
Lysosomal and vesicular proteins	*LAMP1, LAMP2, NPC2, HEXB, GNS, GLB1, GLA, ARSB, PSAP, ERP44, TMEM30A, ASAH1, MOSPD2, FAF2, MLEC*	Lysosomal membrane proteins, hydrolases, and ER-associated regulators of vesicle maturation, acidification, and degranulation
Chaperones and protein folding/degradation	*HSP90AA1, HSP90AB1, HSPA6, DNAJC3, DNAJC13, VCP, PSMD1, PSMD6, PSMD11, PSMD12, PSMB7*	Heat shock proteins, proteasome subunits, protein folding and clearance machinery
Cytoskeleton and trafficking regulators	*ACTR2, ARPC5, RAC1, RHOA, ROCK1, IQGAP1, IQGAP2, DIAPH1, GSN, VCL, ARMC8, CKAP4, GCA, SDCBP, CAB39, CMTM6, CPNE3, DYNLT1*	Actin and microtubule remodeling, with scaffolds and adaptors that direct vesicle trafficking and neutrophil degranulation
Small GTPases and vesicle transport	*RAB7A, RAB6A, RAB10, RAB27A, RAB31, RAP1A, VAPA, COPB1, GDI2, LAMTOR3, TOM1, VPS35L, SCAMP1, SNAP23, IST1, DOCK2, CANT1*	Membrane trafficking, endosome/lysosome fusion, exocytosis
Proton/ion transport and vesicle acidification	*ATP6V1D, ATP6AP2, ATP6V0A1, ATP11A, ATP11B*	Proton pumps and lipid flippases that acidify vesicles and regulate membrane remodeling, essential for lysosomal fusion and degranulation
Metabolic enzymes	*PKM, IDH1, PRDX6, PYGL, ALDH3B1, FABP5, ADA2, STBD1, PDXK, SLC2A3, DEGS1*	Energy metabolism, redox balance, glucose/glycogen/lipid metabolism in activated neutrophils
Complement and innate effectors	*CFP, FCN1, LYZ, CRISPLD2, FGL2, GRN, LRG1, CXCL1, CYBB*	Complement system and neutrophil granule proteins
Apoptosis and stress response	*APAF1, PSEN1, NCSTN, XRCC5, XRCC6, TNFAIP6, PTAFR, PLEKHO2*	Apoptotic regulators, DNA damage repair, stress signaling
Other regulators	*TIMP2, PLAUR, PAFAH1B2, HEBP2, GLIPR1, CCT8, BST1, SLC44A2, KPNB1, MVP, CPPED1, DDX3X*	Diverse regulators including matrix inhibitors, heme-binding proteins, signaling adaptors, and transporters with roles in neutrophil activation

#### Peripheral blood RNA sequencing pathway analysis: downregulated GO and Reactome pathways

The most downregulated GO pathway was ‘structural constituent of ribosome' (Figure 2A), enriched by mitochondrial large (*MRPL*) and small (*MRPS*) ribosomal subunit genes, cytosolic large (*RPL*) and small (*RPS*) ribosomal subunit genes, and other ribosomal-associated proteins (*RPLP* and others; Table 3). The most downregulated Reactome pathway was ‘eukaryotic translation elongation' (Figure 2B), which was also enriched by *RPL, RPS*, and *RPLP* genes, as well as translation elongation factors (*EEF* genes; Supplementary Table 1). In both GO and Reactome, other downregulated pathways were related to mitochondrial function and translational machinery (Figures 2A, B, Figure 3). In Reactome analysis, ‘DNA methylation' was also downregulated (Figure 2B), enriched by DNA methyltransferases (*DNMT1, DNMT3A*; DNA ‘writers') and core histones (*H3, H4, H2B, H2A* families), indicating altered chromatin structure and accessibility (Figure 3).

**Table 3:** The top downregulated Gene Set Enrichment Analysis (GSEA) Gene Ontology pathway was ‘structural constituent of ribosome' (FDR < 0.05). It was enriched by 79 genes, which can be categorized into functional subclusters as follows.

Functional category	Genes	Main role
Mitochondrial large ribosomal subunit (MRPL)	*MRPL4, MRPL17, MRPL37, MRPL49, MRPL57, MRPL21, MRPL28, MRPL39, MRPL46, MRPL55, MRPL32, MRPL18, MRPL10, MRPL9, MRPL12, MRPL52, MRPL11, MRPL24, MRPL43, MRPL41, MRPL34, MRPL14, MRPL54, MRPL20, MRPL36, MRPL2*	Components of the 39S mitochondrial ribosome large subunit
Mitochondrial small ribosomal subunit (MRPS)	*MRPS2, MRPS7, MRPS16, MRPS18A, MRPS18B, MRPS21, MRPS24, MRPS25, MRPS34*	Components of the 28S mitochondrial ribosome small subunit
Mitochondrial ribosomal proteins	*DAP3 (also known as MRPS29)*	Mitochondrial apoptosis-inducing factor, also part of small subunit
Cytosolic large ribosomal subunit (60S, RPL family)	*RPL4, RPL7L1, RPL35A, RPL37A, RPL15, RPL27, RPL12, RPL29, RPL36, RPL23A, RPLP2, RPL39, RPL32, RPL10A, RPL18, RPL41, RPL13A, RPL8, RPL19, RPL35, RPL13, RPLP0, RPL10, RPL3, RPL18A*	Components of the 60S ribosome large subunit, mediating peptide bond formation and elongation
Cytosolic small ribosomal subunit (40S, RPS family)	*RPS3, RPS5, RPS7, RPS10, RPS11, RPS12, RPS14, RPS15, RPS15A, RPS16, RPS18, RPS19, RPS21, RPS27, RPS28, RPS29*	Components of the 40S ribosome small subunit, important for mRNA binding and decoding
Cytosolic acidic ribosomal proteins (RPLP family)	*RPLP0, RPLP1, RPLP2*	Acidic phosphoproteins; regulate elongation factor binding and ribosome activity
Other ribosomal- associated proteins	*FAU, UBA52, RPSA*	FAU = ribosomal protein S30 fusion with ubiquitin-like protein; UBA52 = ribosomal protein L40 fused to ubiquitin; RPSA = laminin receptor with ribosomal origin

#### Comparison between peripheral blood and Tourette syndrome brain transcriptome

To assess convergence between peripheral and brain transcriptomic signatures, we compared the 4,169 blood DEGs from this study with 153 DEGs (FDR < 0.05) previously identified in Tourette syndrome brain tissue (45). We identified 30 overlapping genes between the two datasets (Table 4). Of these, 20 genes were upregulated in both the blood and brain, primarily encoding immune receptors and signaling molecules (*TNFRSF1B, ICAM1, CLEC4E, FCER1G*), cytoskeletal and adhesion regulators (*LCP1, WAS, CD93*), and transcriptional or stress-response factors (*BCL3, SAMHD1, HMOX1;* Table 4). These shared genes highlight overlapping immune and inflammatory mechanisms across peripheral and brain compartments. The single concordant downregulated gene (*SETBP1*) encodes a chromatin-associated transcriptional regulator previously linked to neurodevelopmental syndromes. Nine genes showed opposite directions of regulation (downregulated in blood but upregulated in brain). These were functionally diverse, spanning mitochondrial metabolism (*UCP2*), translation (*RPS11*), cytoskeletal organization (*MYO1G, SYNPO*), and RNA processing (*RNASEH2C, LSM10;* Table 4).

**Table 4:** Overlap of differentially expressed genes (FDR < 0.05) between peripheral blood and Tourette syndrome brain transcriptome analysis. Of total 30 shared DEGs, 20 genes were upregulated, and one gene was downregulated in both the blood and brain analysis. Nine genes were downregulated in the blood and upregulated in the brain.

Functional category	Genes	Main role
Upregulated in both cohorts (20 genes)		
Immune receptors and signaling molecules	*TNFRSF1B, ICAM1, CLEC4E, FCER1G, LRRC25*	Cell-surface and adaptor proteins mediating innate and adaptive immune activation via TNF, Fc-receptor, and lectin pathways
Leukocyte adhesion and migration	*LCP1, WAS, CD93, PRAM1, LAPTM5, SECTM1*	Cytoskeletal and adhesion regulators controlling leukocyte activation, trafficking, and degranulation
Transcription and stress response	*BCL3, FOSL2, SAMHD1, HMOX1, YBX3*	Oxidative-stress, DNA-metabolism, and transcriptional regulators linking inflammatory and stress-response pathways
Signaling and trafficking adaptors	*GNA15, APBB1IP, SNX20*	Intracellular signaling intermediates coupling receptors to downstream cascades and vesicular trafficking
15.6-7.5,-14.1499ptMetabolic and extracellular matrix regulators	*CHSY1*	Enzyme mediating glycosaminoglycan biosynthesis and extracellular-matrix remodeling
Downregulated in both cohorts (1 gene)		
Chromatin/transcriptional regulation	*SETBP1*	Chromatin-associated transcriptional regulator; pathogenic variants cause neurodevelopmental syndromes
Direction mismatch (9 genes) (downregulated in blood and upregulated in brain)		
Mitochondrial metabolism and stress response	*UCP2, TOR3A*	Mitochondrial membrane and ER-stress regulators influencing cellular energy balance
Translation and RNA processing	*RPS11, LSM10*	Ribosomal and spliceosomal components involved in mRNA processing and protein synthesis
Cytoskeleton and trafficking	*MYO1G, KIF19, SYNPO*	Actin- and microtubule-associated proteins essential for vesicle transport and synaptic organization
Cell signaling regulators	*RASAL3*	Ras-GTPase regulator modulating intracellular signaling pathways
Nucleic acid metabolism and stability	*RNASEH2C*	Enzyme maintaining DNA/RNA integrity and genome stability

## Discussion

Gene regulation is increasingly recognized as a central mechanism in neurodevelopmental disorders (NDDs), including tic disorders. In this study, we used bulk RNA sequencing of peripheral blood to investigate common molecular pathways associated with tics and Tourette syndrome. We identified a transcriptomic signature defined by upregulation of chromatin- and cohesin-related pathways, immune activation, and cell signaling, alongside downregulation of translational machinery, mitochondrial function, and DNA methylation in our cohort of tics/Tourette syndrome (Figure 3). We interpret these findings as supporting an altered chromatin state that is associated with gene dysregulation. Similar patterns have emerged in our previous work on both monogenic chromatin syndromes (Kabuki, CHARGE) and non-monogenic conditions such as autistic regression (55–57). In PANS, we have likewise observed dysregulated chromatin, ribosomal, and immune pathways, with single-cell RNA sequencing revealing variation between immune cell types (58–60). Together, the reproducibility of our findings across NDD contexts suggests chromatin and epigenetic dysregulation is an important feature of NDDs.

**Figure 4:**
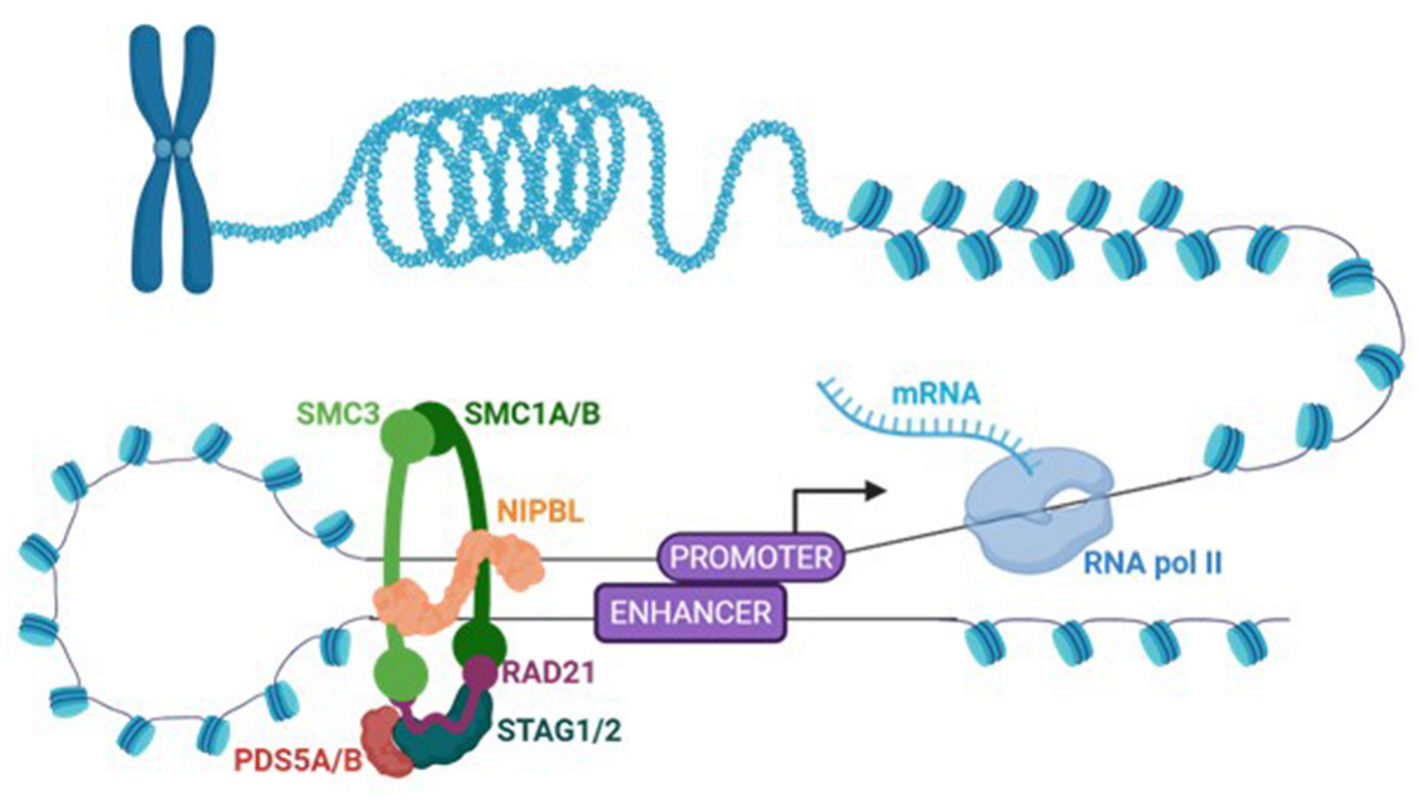
Cohesin is a master regulator of gene transcription. Cohesin is a ring-shaped protein complex formed by *SMC1, SMC3, RAD21*, and a *STAG* subunit. Cohesin organizes chromatin in three dimensions. Cohesin loaders (e.g. *NIPBL*) and regulators (e.g., *PDS5B*) load the cohesin complex onto DNA. By extruding chromatin loops, cohesin brings distant enhancers and promoters into contact to enable gene transcription by RNA polymerase II (RNA pol II). Figure created in BioRender.

A particularly novel finding in our cohort was the enrichment of cohesin-related genes, which have not previously been profiled in tic disorders. Cohesin is a ring-shaped protein complex (*SMC1, SMC3, RAD21*, and a *STAG* subunit; Figure 4) with dual roles in cell biology: it holds sister chromatids together during cell division and organizes chromatin in three dimensions during interphase. By extruding chromatin loops, cohesin brings distant enhancers and promoters into contact (Figure 4), shaping 3D genome architecture and enabling precise transcriptional control (61, 62). Disruption of cohesin or its regulators causes “cohesinopathies” such as Cornelia de Lange Syndrome, marked by intellectual disability, dysmorphic features, and behavioral difficulties, most often due to mutations in *NIPBL* (cohesin loading factor; Figure 4) or, less commonly, *SMC1A, SMC3*, or *RAD21* (63). In neurons, cohesin is particularly important for activity-dependent transcription and synaptic plasticity, with depletion in cortical neurons leading to widespread misexpression of synaptic and cognitive genes, which can be rescued by re-expression of cohesin (63, 64). Together, these findings establish cohesin as a potential regulator of gene expression and neurodevelopment in tics/Tourette syndrome, linking its dysregulation to chromatin-based control of transcription and broader epigenetic regulation.

The immune activation we observed is supported by extensive evidence linking immune dysregulation to tic disorders (4, 23, 24). Enrichment of neutrophil degranulation, interferon signaling, and inflammatory processing pathways in our cohort aligns with hypotheses of a proinflammatory state and may explain why infections and immune triggers exacerbate tics in some individuals (22, 25–28). These findings are consistent with transcriptomic studies of Tourette syndrome brain tissue showing upregulated inflammatory and microglial activation pathways (42, 45, 65). We compared our blood DEGs with those reported in Tourette syndrome brains and identified 30 overlapping genes (Table 4). Most were concordantly upregulated in both tissues, including immune receptors and signaling molecules, cytoskeletal and adhesion regulators, and stress-response factors. The single concordant downregulated gene (*SETBP1*) and several directionally discordant genes were involved in chromatin regulation, mitochondrial metabolism, and translation, suggesting that similar pathways are engaged across tissues, though their transcriptional directionality may differ. Together, these results support a degree of molecular continuity between the brain and blood and reinforce the biological relevance of the peripheral chromatin–immune signature identified in this study.

Mitochondrial dysfunction and ribosomal downregulation situate our findings within the wider NDD landscape. Impaired bioenergetics and oxidative phosphorylation have long been reported across autism, ADHD, and OCD, suggesting shared cellular vulnerabilities (66–68). Similarly, ribosomopathies highlight how defects in ribosome biogenesis produce systemic and neurodevelopmental phenotypes (69), and ribosomal insufficiency has been linked to secondary mitochondrial stress (70). Our data extend these observations and align with striatal findings of mitochondrial dysfunction and impaired oxidative metabolism in Tourette syndrome (65), supporting shared bioenergetic and translational vulnerabilities.

Chromatin regulation plays a pivotal role in brain development by coordinating neurogenesis, synaptic plasticity, and the temporal precision of gene expression programs (71, 72). Genetic studies across NDDs consistently implicate chromatin regulators, translational machinery, and epigenetic factors (41, 73, 74). In our cohort, the convergence of upregulated chromatin and cohesin pathways with downregulation of DNA methylation and translational machinery highlights chromatin as a central “hub” through which gene regulation is disrupted. Complementary evidence from Tourette syndrome brain tissue demonstrates altered chromatin–gene interactions (65). The downregulated DNA methylation pathway, enriched by DNA methyltransferases (*DNMT1, DNMT3A*) and core histones (*H2A, H2B, H3, H4*), further suggests reduced methyltransferase activity and altered nucleosome structure, both of which may impair transcriptional control.

Together, these findings support a model in which disrupted chromatin organization and methylation underpin the downstream transcriptional, ribosomal, and immune dysregulation observed in tics/Tourette syndrome, which may be a common feature of NDDs (55, 56, 58, 59). We propose that chromatin functions as a dynamic interface between genetic predisposition and environmental exposure: variation in chromatin-related genes, combined with environmental factors such as maternal inflammation, stress, or infection, may converge to alter epigenetic control and reprogram neurodevelopmental trajectories (36, 38, 39). Defining these chromatin–immune pathways in peripheral blood provides a foundation for biomarker discovery and future patient stratification in clinical studies.

From a translational perspective, our findings highlight new therapeutic opportunities targeting chromatin and immune dysregulation in tics/Tourette syndrome. Chromatin state is dynamic and druggable. Epigenetic therapies, including histone deacetylase (HDAC) inhibitors, such as butyrate, and ketogenic diet interventions, may “open” chromatin and restore gene regulation (55, 57). In addition, we have shown that intravenous immunoglobulin (IVIG), an immunomodulatory therapy, has effects on methyltransferases and gene regulation in the context of PANS (58, 59). Targeting both chromatin and immune dysregulation could represent a path toward disease-modifying therapies that can optimize neurodevelopmental trajectories for tic disorders, which is an urgent clinical need given the limitations of current symptomatic treatments.

This study has limitations. Bulk RNA sequencing averages signals across immune cell types and cannot resolve cell type–specific drivers of dysregulation. As we have previously shown, single-cell RNA sequencing provides greater granularity, uncovering cell-type variation in gene expression (59). A further limitation is that blood may not directly mirror brain molecular changes, though it remains an accessible and informative tissue reflecting systemic immune and regulatory processes. The age of the post-mortem brain cohort was greater than our blood cohort. Ongoing medication use, comorbidities, and different stages of disease in our cohort may have also influenced gene expression changes. We also note that our sample size is small.

Future research in larger cohorts combining single-cell transcriptomics, chromatin accessibility (ATAC-Seq), histone modifications, DNA methylation, proteomics, and brain-derived data will be essential to refine mechanistic understanding. Functional immune testing is required to assess the immune response in individuals with tics/Tourette syndrome. Longitudinal studies will also help determine the stability of this signature over time, revealing how these transcriptomic changes evolve with disease progression and treatment.

In conclusion, we provide transcriptomic evidence of gene dysregulation in children with tic disorders, characterized by alterations in chromatin cohesion, immune activation, and translational machinery. Taken together, our findings position chromatin biology as a central nexus where genetic risk and environmental exposures converge in tic disorders and associated NDDs. By defining this chromatin–immune signature, we establish a foundation for biomarker discovery and mechanistic targets that connect molecular dysregulation to clinical outcomes, highlighting epigenetic and immune-targeting therapies as promising avenues for disease modification in tics/Tourette syndrome.

## Data Availability

The datasets presented in this study can be found in the GEO repository (accession number: GSE310998).

## References

[1] Ueda K Black KJ. A comprehensive review of tic disorders in children. J Clin Med. (2021) 10:2479. doi: 10.3390/jcm10112479 34204991 PMC8199885

[2] American Psychiatric Association. Diagnostic and Statistical Manual of Mental Disorders. 5th ed. Washington, DC: American Psychiatric Association (2013). doi: 10.1176/appi.books.9780890425596

[3] Cross-Disorder Group of the Psychiatric Genomics Consortium. Electronic address pmhe, cross-disorder group of the psychiatric genomics c. genomic relationships, novel loci, and pleiotropic mechanisms across eight psychiatric disorders. Cell. (2019) 179:1469–82 e11. doi: 10.1016/j.cell.2019.11.020 PMC7077032 31835028

[4] Takahashi N Kato H Nawa Y Ogawa S Tsuchiya KJ Okada T. The role of inflammation in the development of tic symptoms in subjects with adhd. Brain Behavior Immunity Health. (2025) 45:100981. doi: 10.1016/j.bbih.2025.100981 40231211 PMC11994945

[5] Hirschtritt ME Lee PC Pauls DL Dion Y Grados MA Illmann C . Lifetime prevalence, age of risk, and genetic relationships of comorbid psychiatric disorders in tourette syndrome. JAMA Psychiatry. (2015) 72:325–33. doi: 10.1001/jamapsychiatry.2014.2650 25671412 PMC4446055

[6] Sukhodolsky DG Scahill L Zhang H Peterson BS King RA Lombroso PJ . Disruptive behavior in children with tourette's syndrome: association with adhd comorbidity, tic severity, and functional impairment. J Am Acad Child Adolesc Psychiatry. (2003) 42:98–105. doi: 10.1097/00004583-200301000-00016 12500082

[7] Kalyva E Kyriazi M Vargiami E Zafeiriou DI. A review of co-occurrence of autism spectrum disorder and tourette syndrome. Res Autism Spectr Disord. (2016) 24:39–51. doi: 10.1016/j.rasd.2016.01.007

[8] Johnson KA Worbe Y Foote KD Butson CR Gunduz A Okun MS. Tourette syndrome: clinical features, pathophysiology, and treatment. Lancet Neurol. (2023) 22:147–58. doi: 10.1016/S1474-4422(22)00303-9 36354027 PMC10958485

[9] Church JA Fair DA Dosenbach NUF Cohen AL Miezin FM Petersen SE . Control networks in paediatric tourette syndrome show immature and anomalous patterns of functional connectivity. Brain. (2009) 132:225–38. doi: 10.1093/brain/awn223 18952678 PMC2638693

[10] Mataix-Cols D Isomura K Pérez-Vigil A Chang Z Rück C Larsson KJ . Familial risks of tourette syndrome and chronic tic disorders. A population-based cohort study. JAMA Psychiatry. (2015) 72:787–93. doi: 10.1001/jamapsychiatry.2015.0627 26083307

[11] O'Rourke JA Scharf JM Yu D Pauls DL. The genetics of tourette syndrome: a review. J Psychosom Res. (2009) 67:533–45. doi: 10.1016/j.jpsychores.2009.06.006 19913658 PMC2778609

[12] Sundaram SK Huq AM Wilson BJ Chugani HT. Tourette syndrome is associated with recurrent exonic copy number variants. Neurology. (2010) 74:1583–90. doi: 10.1212/WNL.0b013e3181e0f147 20427753 PMC2876824

[13] Georgitsi M Willsey AJ Mathews CA State M Scharf JM Paschou P. The genetic etiology of tourette syndrome: large-scale collaborative efforts on the precipice of discovery. Front Neurosci. (2016) 10:351. doi: 10.3389/fnins.2016.00351 27536211 PMC4971013

[14] Scharf JM Yu D Mathews CA Neale BM Stewart SE Fagerness JA . Genome-wide association study of tourette's Syndrome. Mol Psychiatry. (2013) 18:721–8. doi: 10.1038/mp.2012.69 22889924 PMC3605224

[15] Tsetsos F Yu D Sul JH Huang AY Illmann C Osiecki L . Synaptic processes and immune-related pathways implicated in tourette syndrome. Transl Psychiatry. (2021) 11:56. doi: 10.1038/s41398-020-01082-z 33462189 PMC7814139

[16] Martino D Zis P Buttiglione M. The role of immune mechanisms in tourette syndrome. Brain Res. (2015) 1617:126–43. doi: 10.1016/j.brainres.2014.04.027 24845720

[17] Comings DE. Clinical and molecular genetics of adhd and tourette syndrome. Ann N Y Acad Sci. (2001) 931:50–83. doi: 10.1111/j.1749-6632.2001.tb05773.x 11462757

[18] Brander G Rydell M Kuja-Halkola R Fernández de la Cruz L Lichtenstein P Serlachius E . Perinatal risk factors in tourette's and chronic tic disorders: a total population sibling comparison study. Mol Psychiatry. (2018) 23:1189–97. doi: 10.1038/mp.2017.31 28348386 PMC5984087

[19] Chao T-K Hu J Pringsheim T. Prenatal risk factors for tourette syndrome: a systematic review. BMC Pregnancy Childbirth. (2014) 14:53. doi: 10.1186/1471-2393-14-53 24479407 PMC4015943

[20] Hoekstra PJ Dietrich A Edwards MJ Elamin I Martino D. Environmental factors in tourette syndrome. Neurosci Biobehav Rev. (2013) 37:1040–9. doi: 10.1016/j.neubiorev.2012.10.010 23092654

[21] Han VX Patel S Jones HF Nielsen TC Mohammad SS Hofer MJ . Maternal acute and chronic inflammation in pregnancy is associated with common neurodevelopmental disorders: a systematic review. Transl Psychiatry. (2021) 11:71. doi: 10.1038/s41398-021-01198-w 33479207 PMC7820474

[22] Jones HF Han VX Patel S Gloss BS Soler N Ho A . Maternal autoimmunity and inflammation are associated with childhood tics and obsessive-compulsive disorder: transcriptomic data show common enriched innate immune pathways. Brain Behav Immun. (2021) 94:308–17. doi: 10.1016/j.bbi.2020.12.035 33422639

[23] Hsu CJ Wong LC Lee WT. Immunological dysfunction in tourette syndrome and related disorders. Int J Mol Sci. (2021) 22:853. doi: 10.3390/ijms22020853 33467014 PMC7839977

[24] Wu X Hao J Jiang K Wu M Zhao X Zhang X. Neuroinflammation and pathways that contribute to tourette syndrome. Ital J Pediatr. (2025) 51:63. doi: 10.1186/s13052-025-01874-3 40022157 PMC11871796

[25] Martino D Chiarotti F Buttiglione M Cardona F Creti R Nardocci N . The relationship between group a streptococcal infections and tourette syndrome: a study on a large service-based cohort. Dev Med Child Neurol. (2011) 53:951–7. doi: 10.1111/j.1469-8749.2011.04018.x 21679362

[26] Mell LK Davis RL Owens D. Association between streptococcal infection and obsessive-compulsive disorder, tourette's syndrome, and tic disorder. Pediatrics. (2005) 116:56–60. doi: 10.1542/peds.2004-2058 15995031

[27] Singer HS Giuliano JD Zimmerman AM Walkup JT. Infection: a stimulus for tic disorders. Pediatr Neurol. (2000) 22:380–3. doi: 10.1016/S0887-8994(00)00131-4 10913730

[28] Conelea CA Woods DW. The influence of contextual factors on tic expression in tourette's syndrome: a review. J Psychosom Res. (2008) 65:487–96. doi: 10.1016/j.jpsychores.2008.04.010 18940379

[29] Swedo SE Leonard HL Garvey M Mittleman B Allen AJ Perlmutter S . Pediatric autoimmune neuropsychiatric disorders associated with streptococcal infections: clinical description of the first 50 cases. Am J Psychiatry. (1998) 155:264–71. doi: 10.1176/ajp.155.2.264 9464208

[30] Swedo SE Seidlitz J Kovacevic M Latimer ME Hommer R Lougee L . Clinical presentation of pediatric autoimmune neuropsychiatric disorders associated with streptococcal infections in research and community settings. J Child Adolesc Psychopharmacol. (2015) 25:26–30. doi: 10.1089/cap.2014.0073 25695941 PMC4340334

[31] Chang K Frankovich J Cooperstock M Cunningham MW Latimer ME Murphy TK . Clinical evaluation of youth with pediatric acute-onset neuropsychiatric syndrome (pans): recommendations from the 2013 pans consensus conference. J Child Adolesc Psychopharmacol. (2015) 25:3–13. doi: 10.1089/cap.2014.0084 25325534 PMC4340805

[32] Swedo S. From research subgroup to clinical syndrome: modifying the pandas criteria to describe pans (pediatric acute-onset neuropsychiatric syndrome). Pediatr Ther. (2012) 2:113. doi: 10.4172/2161-0665.1000113

[33] Li Y Wang X Yang H Li Y Gui J Cui Y. Profiles of proinflammatory cytokines and t cells in patients with tourette syndrome: a meta-analysis. Front Immunol. (2022) 13:843247. doi: 10.3389/fimmu.2022.843247 35693824 PMC9177955

[34] Madhusudan N Cavanna AE. The role of immune dysfunction in the development of tics and susceptibility to infections in tourette syndrome: a systematic review. Basal Ganglia. (2013) 3:77–84. doi: 10.1016/j.baga.2013.03.001

[35] Kutuk MO Tufan AE Kilicaslan F Gokcen C Aksu GG Yektas C . Cytokine expression profiles in children and adolescents with tic disorders. Sci Rep. (2024) 14:15101. doi: 10.1038/s41598-024-62121-z 38956051 PMC11219894

[36] Bale TL. Epigenetic and transgenerational reprogramming of brain development. Nat Rev Neurosci. (2015) 16:332–44. doi: 10.1038/nrn3818 25921815 PMC7064155

[37] Millan MJ. An epigenetic framework for neurodevelopmental disorders: from pathogenesis to potential therapy. Neuropharmacology. (2013) 68:2–82. doi: 10.1016/j.neuropharm.2012.11.015 23246909

[38] Oldenburg KS O'Shea TM Fry RC. Genetic and epigenetic factors and early life inflammation as predictors of neurodevelopmental outcomes. Semin Fetal Neonatal Med. (2020) 25:101115. doi: 10.1016/j.siny.2020.101115 32444251 PMC7363586

[39] Weber-Stadlbauer U. Epigenetic and transgenerational mechanisms in infection-mediated neurodevelopmental disorders. Transl Psychiatry. (2017) 7:e1113-e. doi: 10.1038/tp.2017.78 28463237 PMC5534947

[40] Grewal SIS Moazed D. Heterochromatin and epigenetic control of gene expression. Science. (2003) 301:798–802. doi: 10.1126/science.1086887 12907790

[41] Gabriele M Lopez Tobon A D'Agostino G Testa G. The chromatin basis of neurodevelopmental disorders: rethinking dysfunction along the molecular and temporal axes. Prog Neuropsychopharmacol Biol Psychiatry. (2018) 84:306–27. doi: 10.1016/j.pnpbp.2017.12.013 29309830

[42] Lennington JB Coppola G Kataoka-Sasaki Y Fernandez TV Palejev D Li Y . Transcriptome analysis of the human striatum in tourette syndrome. Biol Psychiatry. (2016) 79:372–82. doi: 10.1016/j.biopsych.2014.07.018 25199956 PMC4305353

[43] Lisboa BCG Oliveira KC Tahira AC Barbosa AR Feltrin AS Gouveia G . Initial findings of striatum tripartite model in ocd brain samples based on transcriptome analysis. Sci Rep. (2019) 9:3086. doi: 10.1038/s41598-019-38965-1 30816141 PMC6395771

[44] Voineagu I Wang X Johnston P Lowe JK Tian Y Horvath S . Transcriptomic analysis of autistic brain reveals convergent molecular pathology. Nature. (2011) 474:380–4. doi: 10.1038/nature10110 21614001 PMC3607626

[45] Alshammery S Patel S Jones HF Han VX Gloss BS Gold WA . Common targetable inflammatory pathways in brain transcriptome of autism spectrum disorders and tourette syndrome. Front Neurosci. (2022) 16:999346. doi: 10.3389/fnins.2022.999346 36590292 PMC9799059

[46] Patel S Han VX Keating BA Nishida H Mohammad S Jones H . Ndd-Echo: a standardised digital assessment tool to capture early life environmental and inflammatory factors for children with neurodevelopmental disorders. Brain Behav Immun Health. (2025) 46:101011. doi: 10.1016/j.bbih.2025.101011 40485664 PMC12140942

[47] Dobin A Davis CA Schlesinger F Drenkow J Zaleski C Jha S . Star: ultrafast universal rna-seq aligner. Bioinformatics. (2013) 29:15–21. doi: 10.1093/bioinformatics/bts635 23104886 PMC3530905

[48] R Development Core Team. R: A Language and Environment for Statistical Computing. Vienna: R Foundation for Statistical computing (2010). Available online at: http://www.R-project.org

[49] Wickham H Averick M Bryan J Chang W McGowan LDA François R . Welcome to the Tidyverse. J Open Source Softw. (2019) 4:1686. doi: 10.21105/joss.01686

[50] Risso D Ngai J Speed TP Dudoit S. Normalization of Rna-seq data using factor analysis of control genes or samples. Nat Biotechnol. (2014) 32:896–902. doi: 10.1038/nbt.2931 25150836 PMC4404308

[51] Ritchie ME Phipson B Wu D Hu Y Law CW Shi W . Limma powers differential expression analyses for rna-sequencing and microarray studies. Nucleic Acids Res. (2015) 43:e47-e. doi: 10.1093/nar/gkv007 25605792 PMC4402510

[52] Subramanian A Tamayo P Mootha VK Mukherjee S Ebert BL Gillette MA . Gene set enrichment analysis: a knowledge-based approach for interpreting genome-wide expression profiles. Proc Nat Acad Sci. (2005) 102:15545–50. doi: 10.1073/pnas.0506580102 16199517 PMC1239896

[53] Reimand J Isserlin R Voisin V Kucera M Tannus-Lopes C Rostamianfar A . Pathway enrichment analysis and visualization of omics data using g: profiler, gsea, cytoscape and enrichmentmap. Nat Protoc. (2019) 14:482–517. doi: 10.1038/s41596-018-0103-9 30664679 PMC6607905

[54] Wu T Hu E Xu S Chen M Guo P Dai Z . Clusterprofiler 4.0: a universal enrichment tool for interpreting omics data. Innovation. (2021) 2:100141. doi: 10.1016/j.xinn.2021.100141 34557778 PMC8454663

[55] Hayes JP Han VX Keating BA Nishida H Tsang E Lau X . Butyrate modifies epigenetic and immune pathways in peripheral mononuclear cells from children with neurodevelopmental disorders associated with chromatin dysregulation. Neurotherapeutics (2025) 23:e00792. doi: 10.1016/j.neurot.2025.e00792 41260988 PMC12976508

[56] Nishida H Han VX Keating BA Zyner KG Gloss B Aryamanesh N . Chromatin, Transcriptional and Immune Dysregulation in Children with Neurodevelopmental Regression. medRxiv. (2025):2025.03.05.25322433. doi: 10.1101/2025.03.05.25322433

[57] Tsang E Han VX Flutter C Alshammery S Keating BA Williams T . Ketogenic diet modifies ribosomal protein dysregulation in Kmt2d Kabuki syndrome. EBioMedicine. (2024) 104:105156. doi: 10.1016/j.ebiom.2024.105156 38768529 PMC11134553

[58] Han VX Alshammery S Keating BA Gloss BS Hofer MJ Graham ME . Epigenetic, ribosomal, and immune dysregulation in paediatric acute-onset neuropsychiatric syndrome. Mol Psychiatry. (2025) 30:5389–404. doi: 10.1101/2025.03.28.25324649 40885847 PMC12532593

[59] Han VX Nishida H Keating BA Gloss BS Lau X Dissanayake R . Iv immunoglobulin is associated with epigenetic, ribosomal, and immune changes in pediatric acute-onset neuropsychiatric syndrome. Neurol Neuroimmunol Neuroinflamm. (2025) 12:e200467. doi: 10.1212/NXI.0000000000200467 40953324 PMC12440302

[60] Keating BA Han VX Nishida H Aryamanesh N Marshall LL Gloss BS . Medicinal cannabis plant extract (Nti164) modifies epigenetic, ribosomal, and immune pathways in paediatric acute-onset neuropsychiatric syndrome. MedRxiv. (2025):2025.06.30.25330605. doi: 10.1101/2025.06.30.25330605 41513541 PMC12976522

[61] Kim Y Yu H. Shaping of the 3d Genome by the Atpase Machine Cohesin. Exp Mol Med. (2020) 52:1891–7. doi: 10.1038/s12276-020-00526-2 33268833 PMC8080590

[62] Solé-Ferran M Losada A. Cohesin in 3d: development, differentiation, and disease. Genes Dev. (2025) 39:679–6. doi: 10.1101/gad.352671.125 40345853 PMC12128871

[63] Weiss FD Calderon L Wang Y-F Georgieva R Guo Y Cvetesic N . Neuronal genes deregulated in cornelia de lange syndrome respond to removal and re-expression of cohesin. Nature Commun. (2021) 12:2919. doi: 10.1038/s41467-021-23141-9 34006846 PMC8131595

[64] Calderon L Weiss FD Beagan JA Oliveira MS Georgieva R Wang Y-F . Cohesin-dependence of neuronal gene expression relates to chromatin loop length. Elife. (2022) 11:e76539. doi: 10.7554/eLife.76539 35471149 PMC9106336

[65] Wang Y Fasching L Wu F Suvakov M Huttner A Berretta S . Interneuron loss and microglia activation by transcriptome analyses in the basal ganglia of tourette disorder. Biol Psychiatry. (2025) 98:260–70. doi: 10.1016/j.biopsych.2024.12.022 39892689 PMC12255533

[66] Pinto Payares DV Spooner L Vosters J Dominguez S Patrick L Harris A . A systematic review on the role of mitochondrial dysfunction/disorders in neurodevelopmental disorders and psychiatric/behavioral disorders. Front Psychiatry. (2024) 15:1389093. doi: 10.3389/fpsyt.2024.1389093 39006821 PMC11239503

[67] Valiente-Pallejà A Torrell H Muntané G Cortés MJ Martínez-Leal R Abasolo N . Genetic and clinical evidence of mitochondrial dysfunction in autism spectrum disorder and intellectual disability. Hum Mol Genet. (2018) 27:891–900. doi: 10.1093/hmg/ddy009 29340697

[68] Khaliulin I Hamoudi W Amal H. The multifaceted role of mitochondria in autism spectrum disorder. Mol Psychiatry. (2025) 30:629–50. doi: 10.1038/s41380-024-02725-z 39223276 PMC11753362

[69] Narla A Ebert BL. Ribosomopathies: human disorders of ribosome dysfunction. Blood. (2010) 115:3196–205. doi: 10.1182/blood-2009-10-178129 20194897 PMC2858486

[70] Zhao Q Sarinay Cenik E. Is mitochondrial function at the heart of ribosome-related diseases? Trends Cell Biol. (2025) 35:815–8. doi: 10.1016/j.tcb.2025.07.007 40866130 PMC12903882

[71] Allis CD Jenuwein T. The molecular hallmarks of epigenetic control. Nat Rev Genet. (2016) 17:487–500. doi: 10.1038/nrg.2016.59 27346641

[72] Yao B Christian KM He C Jin P Ming G-L Song H. Epigenetic Mechanisms in Neurogenesis. Nat Rev Neurosci. (2016) 17:537–49. doi: 10.1038/nrn.2016.70 27334043 PMC5610421

[73] De Rubeis S He X Goldberg AP Poultney CS Samocha K Cicek AE . Synaptic, transcriptional and chromatin genes disrupted in autism. Nature. (2014) 515:209–15. doi: 10.1038/nature13772 25363760 PMC4402723

[74] Lasalle J. Autism genes keep turning up chromatin. OA Autism. (2013) 1:14. doi: 10.13172/2052-7810-1-2-610 24404383 PMC3882126

[75] Bolger AM Lohse M Usadel B. Trimmomatic: a flexible trimmer for Illumina sequence data. Bioinformatics. (2014) 30:2114–20. doi: 10.1093/bioinformatics/btu170 24695404 PMC4103590

