# Supplementary material for: Peripheral blood transcriptomics identifies cohesin–chromatin and immune dysregulation in tics and Tourette syndrome
Source: Front Neurol. 2026 Apr 16;17:1730761. doi: 10.3389/fneur.2026.1730761 (PMC13130223; doi:10.3389/fneur.2026.1730761)
Supplement: Supplementary file 1 [file Table_1.docx]

Supplementary Table 1: The top downregulated Gene Set Enrichment Analysis (GSEA) Reactome pathway was ‘eukaryotic translation elongation’ (FDR <0·05). It was enriched by 45 genes, which can be categorised into functional subclusters as follows:

| **Functional category** | **Genes** | **Main role** |
| --- | --- | --- |
| **Cytosolic Large Ribosomal Subunit (60S, RPL family)** | *RPL3, RPL8, RPL10, RPL10A, RPL12, RPL13, RPL13A, RPL15, RPL18, RPL18A, RPL19, RPL23A, RPL27, RPL29, RPL32, RPL35, RPL36, RPL37A, RPL39, RPL41* | Components of the 60S ribosome large subunit, mediating peptide bond formation and elongation. |
| **Cytosolic Small Ribosomal Subunit (40S, RPS family)** | *RPS3, RPS5, RPS7, RPS10, RPS11, RPS12, RPS14, RPS15, RPS15A, RPS16, RPS18, RPS19, RPS21, RPS27, RPS28, RPS29* | Components of the 40S ribosome small subunit, important for mRNA binding and decoding. |
| **Cytosolic Acidic Ribosomal Proteins (RPLP family)** | *RPLP0, RPLP1, RPLP2* | Acidic phosphoproteins; regulate elongation factor binding and ribosome activity. |
| **Other Ribosomal-Associated Proteins** | *FAU, UBA52, RPSA* | FAU = ribosomal protein S30 fusion with ubiquitin-like protein; UBA52 = ribosomal protein L40 fused to ubiquitin; RPSA = laminin receptor with ribosomal origin. |
| **Translation Elongation Factors (EEF family)** | *EEF1A1, EEF1D, EEF1G* | Subunits of eukaryotic elongation factor 1 (eEF1), which delivers aminoacyl-tRNAs to the ribosome during translation. |
